# Improving reproducibility and validity in measures of chronic disease incidence using large-scale linked data in Australia.

**DOI:** 10.23889/ijpds.v9i5.2888

**Published:** 2024-09-18

**Authors:** Kylie-Ann Mallitt, Jacqueline Stephens, Eleonora Dal Grande, Amandi Hiyare, Michelle Dickson, Allison Jaure, Jonathan Craig

**Affiliations:** 1 University of Sydney; 2 Flinders University

## Objective and Approach

Our study aimed to improve methods for identifying incident chronic kidney disease (CKD), cardiovascular disease (CVD), and diabetes in a young cohort from the Antecedents of Renal Disease in Aboriginal Children (ARDAC) study. We linked 20 state and federal administrative health datasets, including hospital records, Medicare claims, pharmaceutical data, and the National Diabetes Services Scheme (NDSS) data for the first time. This approach facilitated the development of new algorithms for disease detection. Notably, we will make the R code for these algorithms publicly available.

## Results

The analysis, involving 3,758 ARDAC study participants, created clinically robust definitions of CKD, CVD, and diabetes (by subtype) in the Australian linked data context; and revealed that all other data sources inaccurately estimated the incidence of diabetes compared to the NDSS data. 

## Conclusions

This key finding illustrates the discrepancy in diabetes incidence estimates and highlights the value of integrating multiple data sources for the investigation of chronic disease.

## Implications

Hundreds of published studies use data linkage to investigate CVD, CKD, and diabetes in the Australian context. Our novel robust case definitions, and the identified poor estimation of diabetes incidence among young people, underscores the need to critically evaluate linked data sources used to inform health policy and research. By sharing the developed R code, we support ongoing efforts to improve transparency and replicability in health research. Our study refines chronic disease definition methods and contributes to more effective public health strategies, with particular focus on improving outcomes for Indigenous communities.

